# MINOCA and INOCA: Role in Heart Failure

**DOI:** 10.1007/s11897-023-00605-1

**Published:** 2023-05-18

**Authors:** Ana G. Almeida

**Affiliations:** Cardiology, Heart and Vessels Department, University Hospital Santa Maria, Faculty of Medicine of Lisbon University, Av. Prof. Egas Moniz, 1649-028 Lisbon, Portugal

**Keywords:** Heart failure, MINOCA, INOCA, Non-obstructive coronary disease, Prognosis

## Abstract

**Purpose of Review:**

Infarction (MINOCA) and ischaemia (INOCA) with non-obstructive coronary disease are recent non-conventional presentations of coronary syndromes that are increasingly recognised in the clinical arena, particularly with the availability of new cardiovascular imaging techniques. Both are related to heart failure (HF). MINOCA is not associated with benign outcomes, and HF is among the most prevalent events. Regarding INOCA, microvascular dysfunction has also been found to associate with HF, particularly with preserved ejection fraction (HFpEF).

**Recent Findings:**

Regardless of the several aetiologies underlying HF in MINOCA, it is likely related to LV dysfunction, where secondary prevention is not yet clearly established. Regarding INOCA, coronary microvascular ischaemia has been associated to endothelial dysfunction leading ultimately to diastolic dysfunction and HFpEF.

**Summary:**

MINOCA and INOCA are clearly related to HF. In both, there is a lack of studies on the identification of the risk factors for HF, diagnostic workup and, importantly, the appropriate primary and secondary prevention strategies.

## Introduction

Coronary artery disease (CAD) is the most common cause of heart failure (HF) [1•, 2, 3•, 4•], which justifies that the presence of obstructive CAD should be actively suspected and searched as an aetiology underlying HF syndrome. In fact, CAD may be associated with HF both by the contractility dysfunction, left ventricular (LV) remodelling and failure in association to myocardial necrosis as well as by diastolic dysfunction, functional mitral regurgitation and chronic atrial fibrillation. Ischaemic cardiomyopathy is a late evolution of CAD resulting from loss of large areas of myocardial cell loss with possible areas of hibernating myocardium. Besides the risk of HF and hospitalisations, the patients with ischaemic HF are at high risk of arrhythmias, stroke and death [1•, 2, 3•] while they are potentially candidates for early intervention and secondary preventive measures for major adverse events [1•, 2, 3•].

Chronic CAD may also be a cause of HF even in the absence of ischaemic symptomatology, where dysfunctional hibernating myocardium may determine reduced ejection fraction and diastolic dysfunction in association to reduced LV relaxation and increased ventricular wall stiffness [1•].

In the last years, different presentations of ischaemic heart disease have emerged that are beyond the classical obstructive CAD as the underlying aetiology, both acting as potential substrates for the development or worsening of heart failure. Ischaemia and infarction with non-obstructive coronary disease are such presentations and will be covered in this review paper.

Myocardial infarction with non-obstructive coronary artery disease (MINOCA) is a working diagnosis, encompassing a range of conditions where obstructive coronary disease is excluded following a thorough investigation involving non-invasive imaging and invasive techniques that are mandatory for definite clarification and management decisions [5••, 6••].

Ischaemia with non-obstructive coronary artery disease (INOCA) is another newly classified category of ischaemic heart disease, characterised by the presence of angina and/or evidence of myocardial ischaemia in the absence of obstructive coronary disease, most frequently encompassing coronary microvascular disease (CMD) and epicardial coronary spasm [4•]. Both conditions may determine or contribute to myocardial dysfunction in patients with clinical evidence of ischaemia, with microvascular dysfunction playing a major role. This condition acts not only in cases of ischaemia but also in other clinical settings, including non-ischaemic cardiomyopathies, Takotsubo syndrome, heart failure with preserved ejection fraction (HFpEF) and obstructive CAD [7••, 8].

In the following chapters, these two conditions will be presented in their potential role and the involved mechanisms responsible for determining or worsening HF.

## MINOCA

### Definitions and Characterisation

MINOCA describes patients with a diagnosis of acute myocardial infarction (AMI) who are found to have non-obstructive or normal coronary arteries following coronary angiography [5••, 6••].

This entity was first documented by Miller et al. in 1951 from autopsy reports where myocardial necrosis was found to associate to normal coronary arteries [6••]. The availability of cardiovascular diagnosis based on imaging techniques brought light to the understanding of suspected MINOCA, which represents an umbrella over several underlying conditions with varied pathophysiology. Studies from last years have shown that up to 6–15% of patients presenting with the clinical syndrome AMI will be given the working diagnosis of MINOCA [5••, 6••]. Given the heterogeneity of the underlying aetiologies for the suspected MINOCA, a thorough investigation must be pursued since management depends on the identified cause.

MINOCA should be in fact regarded as a working diagnosis and is generally characterised by two sets of criteria. The first criterion consists of the confirmation of AMI according the Fourth Universal Definition of Myocardial Infarction [9]. Second, the absence of coronary lesions in the coronary angiography sufficiently severe to compromise myocardial blood flow, encompassing the complete absence of coronary lesions and the presence of obstructive lesions corresponding to < 50% lumen stenosis (mild stenosis < 30% and moderate from 30 to 50%) [5••, 6••].

Regarding the definition of AMI as a criterion for MINOCA, there is a need for differentiating between myocardial infarction and injury as focused in the recent guidelines on non-ST segment acute coronary syndromes [3•]. Myocardial injury, characterised by raised troponin, is the hallmark of conditions such as myocarditis, cardio-oncologic toxicity, myocardial contusion, allograft rejection or specific cardiomyopathies and should be outside the scope of MINOCA. Takotsubo is also proposed as a condition to be excluded from MINOCA, due to its distinct pathophysiology and the fact that myocardial oedema dominates the picture over myocardial injury typically minimal [10]. Other non-cardiac causes such as pulmonary thromboembolism may be associated with chest pain and raised troponin and should be excluded before suspected MINOCA is proposed [5••, 6••].

An inherent limitation for MINOCA definition concerns the criterion for absence of significant CAD since severity is typically assessed visually using the cut-off of 50% for coronary lesions. Although consistent with previous coronary angiography guidelines, this value is somewhat arbitrary and associates to inter-observer and intra-observer variability, and moreover, intermediate stenosis may correspond to more severe stenosis from a physiological assessment [11].

### Clinical Features and Epidemiology

In comparison with classical AMI, MINOCA patients present typically less commonly ST segment deviations in the ECG and lower increases in cardiac troponin [12]. Women were found to have twice the prevalence of MINOCA in comparison to men (50% vs 25%), and a higher prevalence was also found in Black, Hispanic and Pacific ethnicities, who are more represented in MINOCA in comparison with classical AMI [13, 14•].

### Specific Causes of MINOCA

#### Coronary Atherosclerosis

Up to two-thirds of causes of MINOCA are attributed to atherosclerotic plaque disruption including rupture, erosion and calcific nodules [5••, 6••, 15]. Plaque disruption may occur in areas of the vessel that appear normal on angiography; however, minimal degree of atherosclerosis should be present and usually is seen. Erosion, a more frequent feature in women, is characterised by an intact fibrous cap with a thrombus superimposed. Thromboembolism and microvasospasm may associate. In both cases, intravascular ultrasound (IVUS) or high-resolution imaging with optical coherence tomography (OCT) may be necessary for the diagnosis, suggesting that this aetiology may be underdiagnosed since IVUS and OCT are not used systematically [5••].

#### Coronary Artery Spasm

Coronary vasospasm is characterised as an intense vasoconstriction of an epicardial coronary artery resulting in reduced myocardial blood flow with possible arrhythmias, syncope and transient HF. This condition may occur superimposed on atherosclerotic lesions but more usually is observed in coronaries without lesions. Vasospasm may occur both in response to toxins, drugs or tumours, namely cocaine or phaeochromocytoma, due to vascular smooth muscle hyper-reactivity, or spontaneously due to abnormalities in coronary vasomotor tone and endogenous vasoactive substances [5••]. The role of microvascular spasm is not well clarified and requires further studies [16•].

#### Coronary Thromboembolism

**Fig. 1:**
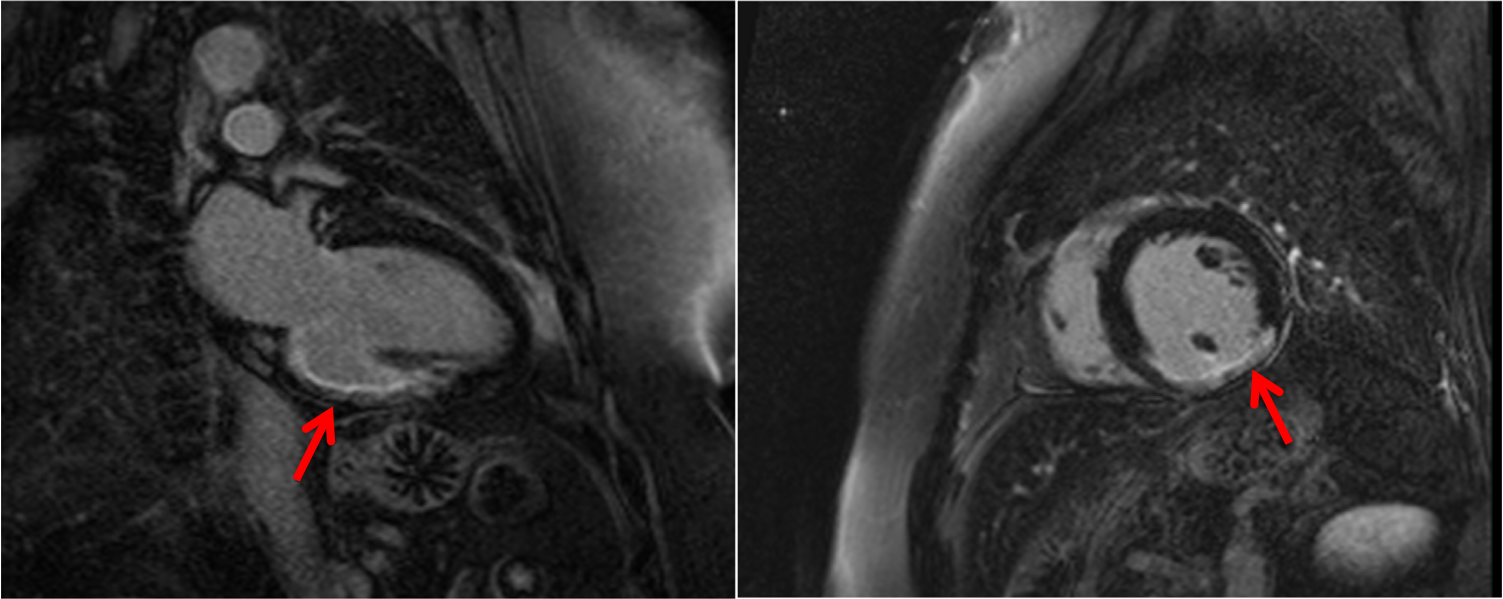
A 65-year-old man with history of hypertension presented with chest pain and ECG with ST segment elevation in DII and aVF leads. Invasive coronary angiography showed non-obstructive coronary disease, and subsequent troponin was raised. Mid-basal Inferior wall was hypokinetic on echocardiography. Cardiac magnetic resonance showed subendocardial late gadolinium enhancement (arrows) at the mid-basal inferior wall confirming an ischaemic pattern from myocardial infarction. A 24-h Holter revealed paroxysmal atrial fibrillation suggesting embolic aetiology. At 3-month follow-up, LV was mildly dilated and the ejection fraction was 38%, with NYHA functional class II

Thromboembolism arising from left atrial appendage and atrium (namely in association with atrial fibrillation), left ventricle, mitral or aortic valves, vegetations, tumours or proximal coronary artery is a possible cause for MINOCA (Fig. 1). Coronary thromboembolism has been found to be associated to MINOCA in up to 2.9% [17, 18]*.* Hypercoagulable states such as pregnancy, autoimmune disorders (antiphospholipid syndrome), heparin-induced thrombocytopenia, thrombotic thrombocytopenic purpura or active malignancy are possible causes for arterial and venous thrombosis [5••, 6••] that must be considered as hypothesis and searched when other causes are absent.

#### Spontaneous Coronary Artery Dissection

Spontaneous coronary artery dissection (SCAD) is caused by the dissection of the coronary arterial wall layers by an intimal flap or intramural haematoma determining coronary obstruction with variable degrees and necrosis. In all acute coronary syndromes, SCAD occurs in 2–4%, with a prevalence of up to 35% in women < 50 year old [19, 20]*.* SCAD is associated with pregnancy, Ehlers-Danlos syndrome and Marfan syndrome, and particularly with fibromuscular dysplasia, where SCAD is the most common cardiac condition. Although conventional coronary angiography may suggest the diagnosis, the use of IVUS or OCT is required for definitive confirmation [20].

#### Supply-Demand Mismatch—Type 2 AMI

This aetiology is characterised by myocardial cell necrosis due to supply–demand mismatch. In addition to at least one of the other criteria for AMI, this type is characterised by significant increase and/or decrease in troponins in the absence of evidence for coronary plaque rupture and stenosis. Causes must determine a profound imbalance of supply-demand and may be tachy or bradyarrhythmia, respiratory failure, hypotension, shock, severe hypertension, heart failure, cardiomyopathy or injurious effects of pharmacological agents (e.g., catecholamines) [5••, 21].

#### MINOCA of Uncertain Aetiology

Although in 8–25% the cause of MINOCA remains undetermined causing uncertainty regarding management [5••], a recent study indicates that cardiac magnetic resonance (CMR) can contribute significantly to further elucidation [22].

### Clinical Investigation

Following that exclusion of non-obstructive coronary disease by coronary angiography and Takotsubo is excluded by echocardiography, MINOCA should be considered, and lead to prompt further investigations in order to ascertain a final diagnosis. It is mandatory to reassess angiographic images, ensuring that obstructive disease has not been overlooked or that IVUS or OCT are not needed for further clarification. CMR is an increasingly key tool in MINOCA patients because, besides confirming the diagnosis of AMI, based on the presence of the typical ischaemic subendocardial pattern of late gadolinium enhancement (LGE), may provide clues for the potential aetiologies [23]. LGE CMR imaging is able to identify currently at least 1 g of infarcted myocardium [24], but in a proportion of patients with MINOCA, there is no evidence of LGE [5••, 6••] suggesting the presence minimal necrosis, under the capacity of detection, or otherwise a broader spatial distribution. Transesophageal echocardiography, cardiac CT angiography, Holter monitoring and hypercoagulation state evaluation are further modalities for aetiology assessment.

### MINOCA and Heart Failure

Outcomes of patients presenting with MINOCA have shown heterogeneity according to different methodologies and populations, depending as well on the underlying cause.

Several studies found that MINOCA patients have better outcomes than the ones with conventional AMI, with lower yearly MACE [25••]. However, more recent data clearly show that MINOCA should not be considered benign since the associated risk for long-term mortality, re-infarction and HF has been shown as significant [14•, 26–30, 31••].

In fact, compared to subjects without apparent acute cardiovascular disease, MINOCA patients had a more than twofold increased risk of MACE with a constantly increasing event rate over time. This was mainly driven by the risks of cardiovascular mortality including the risks of heart failure and recurrent MI.

Heterogeneity in inclusion criteria, study design, the impact of underlying causes of MINOCA patients as well as the inclusion of small cohorts have made challenging assessing the outcomes of these patients as well its pathophysiological basis. Currently, five main aetiologies of MINOCA should be considered, after the exclusion of myocarditis and Takotsubo, according to the latest position paper from the ESC and the Scientific Statement from the AHA [5••, 6••]. However, previous studies also included these last conditions leading to conclusions that cannot be taken together. An important limitation in the outcome assessment is the lack of limited data on cardiovascular morbidity and the causes of mortality in MINOCA [26, 31••], which suggests the needs of a large population and a long follow-up.

Several studies have addressed the outcomes of patients with MINOCA according to its etiological subtypes, although limitations are inherent to this approach, since the broad classification encompasses heterogeneous mechanisms and these may intervene in the prognosis by themselves. This is, for instance, the case of coronary embolism where prothrombotic conditions have different pathophysiological basis from atrial fibrillation to valvular vegetations.

Outcomes in MINOCA are firstly, and most likely, related to the amount, transmurality and location of myocardial infarction, in parallel to the pathophysiology of MI from obstructive coronary disease. Ensuing LV dysfunction and remodelling should be primary players in the prognosis that ultimately may lead to heart failure, arrhythmias and death. In MINOCA patients, the hallmark of necrosis is central and common to every aetiology and this could be one of the most important factors with prognostic impact. CMR is currently able to detect and quantify myocardial necrosis in MINOCA, which is detectable in most cases, helping predicting outcomes and guiding early and timely preventive therapies for LV remodelling evolution [32•].

In the SWEDEHEART Registry (Swedish Web System for Enhancement and Development of Evidence-Based Care in Heart Disease Evaluated According to Recommended Therapies) [30], in more than 9136 patients with MINOCA, the risk of mortality, re-infarction, ischaemic stroke and heart failure at 4.1-year follow-up was 13.4%, 7.1%, 4.3% and 6.4%, respectively. Furthermore in a registry-based study from the TOTAL-AMI [31••], using data from the SWEDEHEART, which included > 7200 patients with MINOCA and 69,276 with first conventional AMI, morbidity and cause-specific mortality were examined at a median follow-up of 3.4 years. While patients with MINOCA had a cardiac mortality rate of 5.3%, they had the highest prevalence of heart failure and 27.6% of those who underwent echocardiography had impaired left ventricular ejection fraction. The risk of MACE among MINOCA patients was driven by the risk of cardiovascular mortality (HR 3.61), recurrent MI (HR 4.09) and heart failure (HR 2.67).

However, most studies have analysed outcomes in a general perspective of the working diagnosis of MINOCA without taking into account both the specific aetiologies and the cause-specific mortality and morbidity.

In an early study from the Korean Acute Myocardial Infarction Registry [33], the authors included prospectively 8510 patients with AMI and found that prognosis was not different between the group with almost normal coronaries and the one with patients with single or double-vessel disease, with 12-month MACE of 7.8% versus 12.2%, *p* = 0.359, with MACE defined as cardiac death, MI and target vessel revascularisation. Both groups showed however a significantly better prognosis than the group of patients with 3 vessels or left main disease. Almost half of the MINOCA patients had an unknown cause, but CMR, IVUS or OCT were rarely used. However, this large study stresses the prognostic impact of MINOCA.

The VIRGO (Variation in Recovery: Role of Gender on Outcomes of Young AMI Patients) study [27] was a prospective observational study of 2690 patients < 55 years, where 11.1% were classified as MINOCA, the majority with no cause identified, with limited use of appropriate etiological evaluation. Similar proportions of patients with MINOCA and classical MI had reduced ejection fraction, or presented with heart failure, which was present in about 5% of MINOCA patients. Patients with MINOCA had similar 1-month and 1-year mortality rates and comparable quality-of-life measures as patients with classical AMI. Importantly, 12-month mortality for MINOCA was 2 times higher than the expected annual mortality for age and sex. Of note, these patients were significantly less likely to undergo secondary prevention medications and cardiac rehabilitation suggesting the lack of guidance in this heterogeneous condition.

The large study ANZACS-QI (All New Zealand Acute Coronary Syndrome—Quality Improvement) registry included 302 from 2070 (15%) patients with non-obstructive coronary artery disease from a cohort of 2070 MI [12, 34]. Compared to patients with obstructive disease, the ones with non-obstructive group were younger (57 versus 61 years), more likely to be women (50% vs 23%) and from Maori or Pacific versus European ethnicity. They were also less likely to receive secondary prevention medications. MINOCA patients had a higher prevalence of normal LV ejection fraction (56.6% vs 43.7%), lower but important rate of heart failure (Killip classes II, III, IV; 5.8% vs 9.4%), as well as hospital death (0.2% vs 1.5%), but whole prevalence of MACE in MINOCA was not negligible. At 2 years of follow-up, recurrent MI was 7% and mortality 4.9%, showing an important long-term risk.

In a recent large registry, Dreyer et al. addressed the outcomes of MINOCA vs. conventional AMI in a large Medicare population, which included 286,760 > 65-year-old patients with STEMI and NSTEMI [35]. At 12-month follow-up, and in comparison with conventional MI, MINOCA patients had lower mortality and MACE (12.3% vs 16.7% and 18.7% vs 27.6%, respectively). However, rates of MACE in MINOCA were significant, with heart failure occurring in 5.9% at 12 months in comparison to 9.3% in conventional AMI.

A 2015 systematic review [14•] has found a prevalence for MINOCA of 6%, while the SWEDEHEART Registry described 8% and the ANZACS-QI found about 12%, translating into an important frequency of MI patients without obstructive coronary artery disease that will likely develop an important rate of MACE in the follow-up.

A recent systematic review [36••] found that the long-term mortality after MINOCA was lower than that in patients with conventional MI, but it was not trivial. Annual rates of long-term total mortality were 2.2% and 5.0% for MINOCA and CAD AMI. Meta-regression analysis showed that normal ejection fraction and normal coronary arteries at angiography were inversely related to long-term mortality, whereas use of beta-blockers during follow-up and ST depression on the admission electrocardiogram were directly related with worse outcome.

The impact of secondary prevention on prognosis of MINOCA has been addressed by few observational studies, but still awaiting randomised studies. In the SWEDHEART registry, there was a significant lower risk of MACE of 23% and 18% for patients treated with statins and ACEI/ARB, respectively [30]. While effects of statins are expected to stabilise non-significant CAD, because plaque ruptures and erosions causing MI may occur from non-significant plaques, the preventive effect of ACEI/ARB on MACE suggests the mechanism of LV dysfunction as an important intervenient in the process, since these therapies should act on remodelling and survival [4•]. A recent observational study [37] found that adverse events risk at 2-year follow-up decreased when statins and ACEI/ARB were used, whereas the risk of adverse events was not lower in patients with aspirin, clopidogrel and β-blocker. Additionally, patients with MINOCA were less likely to receive secondary prevention medications at the time of discharge and more likely to have early discontinuation of medications at the time of follow-up. The influence of prevention using selected secondary preventive measures seems associated with prognostic benefit in patients with MINOCA, in particular achieving target range low-density lipoprotein cholesterol levels [38]. Second, and importantly, the selection of therapies and the influence on outcomes after the secondary prevention programs taking into account the specific aetiology underlying MINOCA are still awaited from randomised studies [5••, 6••].

**Fig. 2:**
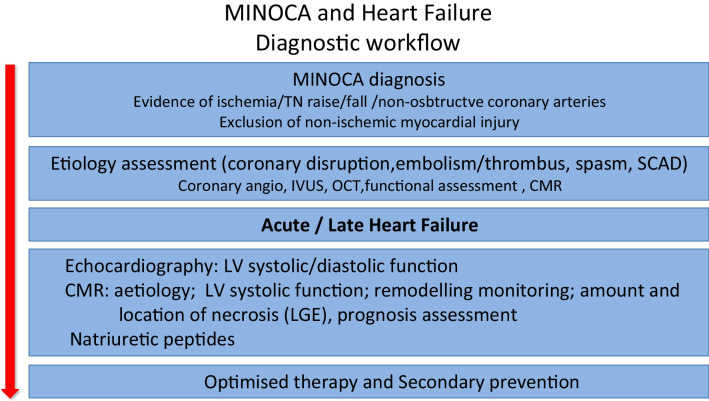
Diagnostic workflow in patients with the diagnosis of MINOCA and heart failure development. TN, troponin; CMR, cardiac magnetic resonance; LV, left ventricle; LGE, late gadolinium enhancement

In summary, MINOCA as a working diagnosis encompassing a number of conditions with heterogeneous mechanisms should be associated with distinct clinical significances, outcomes and management. Multiple studies have shown that, albeit generally associated with a lower mortality and MACE rates than in conventional AMI, MINOCA in its broad perspective is not benign since early and late outcomes are not trivial. HF is among the most prevalent MACE, related to LV dysfunction. A systematic investigation of the conditions underlying the diagnosis must be undertaken in order to more appropriately decide on the management with prognosis likely varying accordingly [5••, 6••]. Among the diagnostic modalities, CMR is especially useful for confirming the presence of myocardial infarction, assessing LV function reliably and helping in the aetiology identification. A proposal for a diagnostic workflow regarding the development of heart failure in association to MINOCA is presented in Fig. 2. Regarding the best therapies, which are less likely to be prescribed, studies are however still scarce, and secondary prevention measures await future scrutiny.

## INOCA

### Introduction and Epidemiology

Angina pectoris, the most common symptom of ischaemic heart disease, affects approximately 112 million people in the world [39•]. However, a large proportion of patients, up to 70%, with angina and evidence of ischaemia undergoing coronary angiography, have no obstructive coronary disease, defined as the presence of > 50% coronary stenosis [4•]. These findings define the specific condition of ischaemia with non-obstructive coronary artery disease (INOCA), which from studies in the last decades encompasses two main pathophysiological mechanisms—coronary microvascular dysfunction (CMD) and epicardial coronary vasospasm [4•, 40•].

Women have at least the double expected prevalence of ischaemia from INOCA as confirmed from coronary angiography in comparison to men*.* In a study of INOCA including patients with stable angina, 70.2% of female versus 43.1% of male patients had coronary microvascular dysfunction (CMD) or epicardial artery vasospasm [41, 42]*.*

This condition is not a benign condition since it was found to associate with an increased long-term risk of adverse clinical events including myocardial infarction, recurrent ischaemia, heart failure, hospitalisations and cardiac death as well as lower quality of life [43–45]. In clinical ground, as stated by the guidelines in chronic coronary syndromes, a discrepancy between findings regarding coronary anatomy, the presence of symptoms, and the results of non-invasive tests frequently occurs [4•]*.* A thorough identification of the involved mechanism must be performed by appropriate diagnostic approaches followed by a decision on the best management strategy. However, studies on the most appropriate clinical management are still scarce and gaps in knowledge remain without full clarification*.*

### Endotypes of INOCA and Pathophysiology

According to current concepts as proposed by the COVADIS group, there are 2 main endotypes of INOCA to consider, coronary microvascular disease (CMD) and epicardial coronary vasospasm [46].

CMD, underlying microvascular angina, is characterised clinically by angina and ischaemia evidence by stress tests. Myocardial ischaemia may result both from structural changes of the microvasculature (microvascular remodelling, microembolisation, smaller calibre of coronaries and the lower vascular density) with reduced conductance, or to vasomotor disorders affecting the coronary arterioles, causing dynamic arteriolar obstruction [47].

Vasospastic angina (VSA) is the clinical manifestation of myocardial ischaemia caused by dynamic epicardial coronary obstruction caused by an epicardial coronary artery spasm. Typically, this angina associates with > 90% constriction with angina and ischaemic ECG changes either spontaneously or in response to a provocative stimulus (typically acetylcholine, ergot or hyperventilation) and with no relationship to effort [48].

### Clinical Diagnosis

#### Coronary Microvascular Disease

Regarding the diagnosis of CMD, the following criteria have been proposed [49]: (a) presence of symptoms and objective evidence of ischaemia; (b) absence of significant coronary disease; and (c) evidence of impaired coronary microvascular function: impaired coronary flow reserve (CFR); abnormal coronary resistance indices; coronary microvascular spasm; and coronary slow flow phenomena. So far, the reference method is the invasive testing of CFR and the index of microvascular resistance (IMR) using acetylcholine and adenosine to assess for endothelial-dependent and endothelial-independent dysfunction [47]. Abnormal values have been < 2.0 for CFR and > 25 units for microvascular resistance. This assessment is however not routinely used in clinical setting, and non-invasive testing could be the preferred if proved accurate.

PET with vasodilator stress is considered the gold standard of non-invasive diagnosis of CMD, with myocardial flow reserve (MFR) validated by invasive assessment [50] and against outcomes, but uses radiation, has limited availability and is costly. Two additional techniques have shown usefulness. Stress Doppler echocardiography may identify the maximal diastolic flow in the left anterior descending coronary artery at rest and during adenosine or dipyridamole stress, in order to estimate CFR. This technique has been validated against intracoronary Doppler measurements and outcomes [51]. Myocardial contrast echocardiography shows a particularly significant potential for CMD detection, but the lack of widespread experience has represented a limitation for its use. Cardiac magnetic resonance (CMR) allows the qualitative diagnosis and a quantitative assessment by measuring the CFR, microcirculatory perfusion index (MPI) and the perfusion resistance index (MPRI), both correlating well with invasive measurements as well as having prognostic impact [52, 53••]. Cut-offs for diagnosis of CMD using the available non-invasive techniques are currently being assessed as well as possible differences between women and men [54].

### Epicardial Coronary Vasospasm

Patients with vasospasm are frequently younger and have fewer cardiovascular risk factors than patients with effort angina [4•, 48].

Diagnosis is based on ST segment elevation on the ECG (or Holter monitoring) during the chest pain episode, but confirmation needs angiographic documentation of coronary spasm using of a provocation test with intracoronary administration of acetylcholine or ergonovine, which have been found safe tests [48].

### INOCA and Heart Failure

CMD seems to precede the development of epicardial lesions, particularly in women [4•] and is associated with impaired outcomes. Several studies have shown that prognosis is associated with abnormal indices of CMD. Among patients with diabetes undergoing diagnostic work-up, those without obstructive epicardial disease but with an abnormal CFR have similarly poor long-term prognosis in comparison to those with obstructive epicardial disease [55]. Moreover, in patients with INOCA, the CFR value obtained during the diagnostic work-up behaved as contiguous predictor of an excess of MACE in the long-term prognosis, particularly in women [56, 57].

**Fig. 3:**
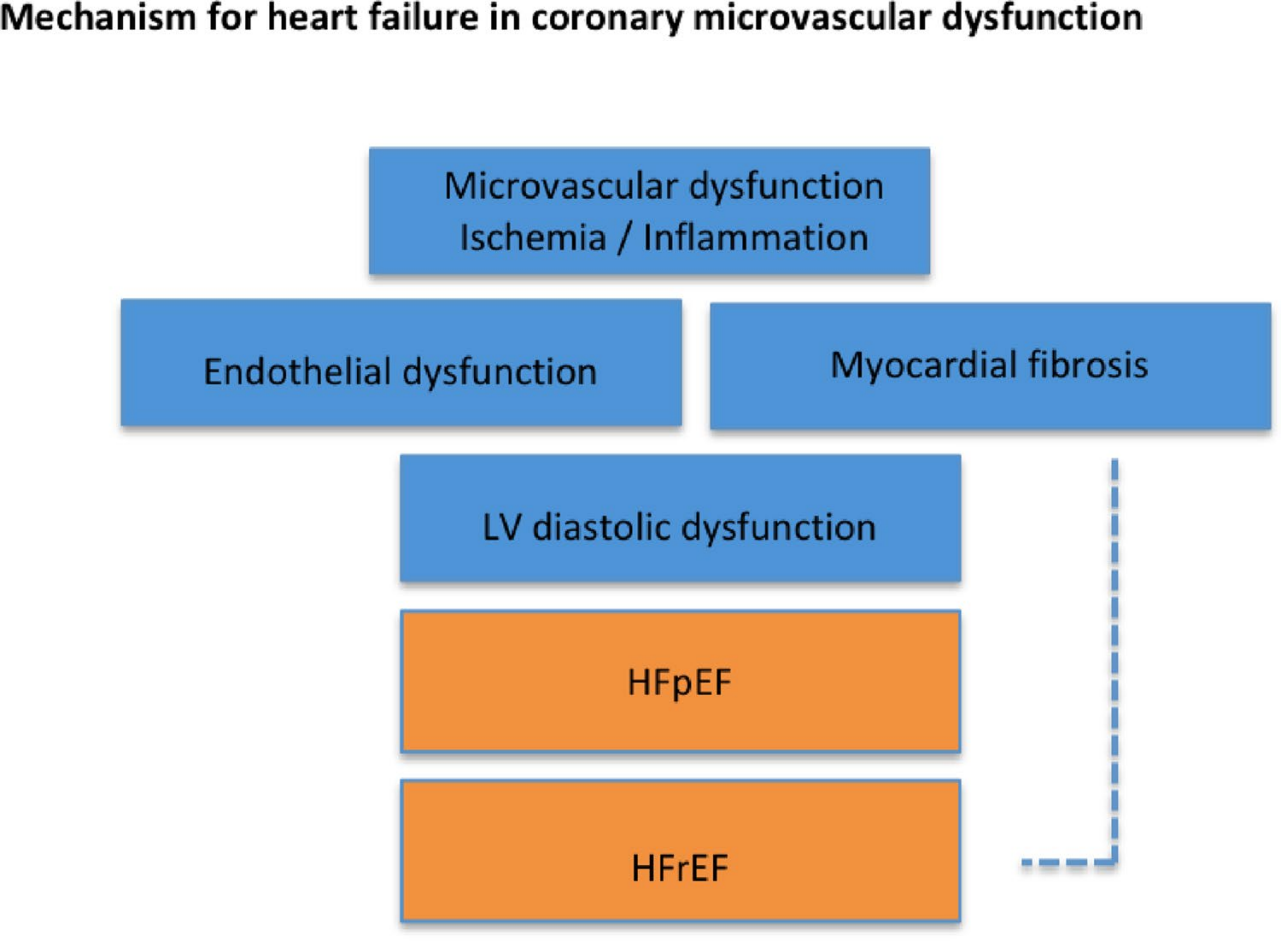
Proposed mechanism for heart failure in INOCA from coronary microvascular disease. LV, left ventricle; HFpEF, heart failure with preserved ejection fraction; HFrEF, heart failure with reduced ejection fraction

Several studies have found an association between CMD indices and increased risk of ventricular dysfunction and heart failure, particularly in the presentation of preserved ejection fraction (HFpEF). Women are particularly affected by CMD, often unrecognised in clinical arena due incomplete diagnostic assessment, since methods and decision algorithms are not yet clearly established [58]. Microvascular ischaemia and ensuing related endothelial dysfunction could lead to heart failure. LV diastolic dysfunction has been found to occur early in the ischaemic cascade in patients with CMD with microvascular dysfunction playing a likely role in the link with HF, namely with preserved ejection fraction [59, 60, 61••]. It is hypothesised that risk factor conditions (hypertension, dyslipidemia, diabetes, oestrogen loss) could promote a pro-inflammatory, pro-oxidative state rendering the coronary microvasculature to dysfunction and the myocardium vulnerable to ischaemia and fibrosis, both leading to HF [3•, 62] (Fig. 3).

In fact, a retrospective study on women with INOCA from the Women’s Ischaemia Syndrome Evaluation (WISE) study, followed for 6 years, showed that hospitalisation from heart failure was the most frequent MACE at follow-up, mostly from HFpEF [63].

More recently, in an analysis from the Coronary Vascular Dysfunction (WISE-CVD) study [64••], in women with impaired CFR, low resting coronary flow velocity measured invasively was associated with higher LV end-diastolic filling pressure, lower LV ejection fraction and abnormalities in late systolic and diastolic strain rates. These changes could contribute to increased risk for adverse outcomes particularly heart failure in women with CMD.

A recent study involved 201 patients with symptoms of ischaemia, positive stress PET and non-obstructive coronary lesions who were followed for 4 years. In adjusted analyses, impaired CFR as representing microvascular ischaemia was independently associated with diastolic dysfunction (echocardiographic *E*/*e′* > 15, OR 2.58, 95% CI 1.22–5.48) and composite cardiovascular outcomes or HFpEF hospitalisation alone (HR 2.47, 95% CI 1.09–5.62). Patients with both impaired CFR and diastolic dysfunction had fivefold increased risk of hospitalisation for HFpEF [59, 65].

**Fig. 4:**
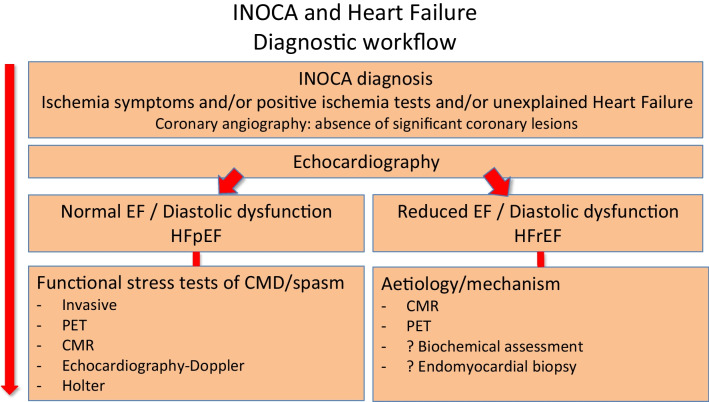
Proposed diagnostic workflow in patients with the diagnosis of INOCA and heart failure development. EF, ejection fraction; HFpEF, heart failure with preserved ejection fraction; HFrEF, heart failure with reduced ejection fraction; CMD, coronary microvascular dysfunction; CMR, cardiac magnetic resonance

In a large retrospective study, Braga et al. [66•] investigated whether the presence of non-obstructive coronary disease in patients with HF with reduced ejection fraction had prognostic impact in comparison to the ones without coronary lesions and obstructive coronary disease. They found that non-obstructive disease was independently associated with an increased hazard of cardiovascular death, non-fatal AMI, non-fatal ischaemic stroke and HF hospitalisations with a rate of all-cause death that is 18% higher compared with those with no apparent CAD. In fact, in patients with MINOCA and non-obstructive lesions, it is proposed that structural and functional disorders in atherosclerosis affect both epicardial coronaries and microcirculation and that CMD is responsible for ischaemia and ensuing burden of heart failure with preserved ejection fraction (HFpEF) [59]. Although data is still lacking for recommendations on the best diagnostic algorithm for diagnosis in suspected INOCA in association to HF, we suggest a diagnostic workflow for this purpose in Fig. 4.

In summary, INOCA has been found to associate to increased risk of ventricular dysfunction and heart failure, particularly HFpEF with women more frequently affected. Endothelial dysfunction from microvascular ischaemia is likely a key mechanism underlying LV diastolic dysfunction as a primary event promoting heart failure.

## Conclusions

MINOCA and INOCA represent non-conventional presentations of myocardial necrosis and ischaemia from non-obstructive coronary disease. Both may be associated with LV dysfunction and heart failure representing major adverse events with impact in the prognosis. MINOCA is a working diagnosis encompassing a number of conditions where a general mortality and MACE rates are lower than in conventional AMI, but late outcomes are not trivial with HF and LV dysfunction among the most prevalent MACE. Regarding INOCA, microvascular ischaemia and ensuing endothelial dysfunction are major links for LV diastolic dysfunction and HF, particularly HFpEF. Heart failure as a major outcome in both conditions must be acknowledged and subject to appropriate management, which awaits further studies for proper clarification.

## Abbreviations

AMI: Acute myocardial infarction
CAD: Coronary artery disease
CFR: Coronary flow reserve
CMD: Coronary microvascular disease
CMR: Cardiac magnetic resonance
HF: Heart failure
HFpEF: Heart failure with preserved ejection fraction
INOCA: Ischaemia with non-obstructive coronary artery disease
IVUS: Intravascular ultrasound
LGE: Late gadolinium enhancement
LV: Left ventricle
MI: Myocardial infarction
MINOCA: Myocardial infarction with non-obstructive coronary artery disease
OCT: Optical coherence tomography
SCAD: Spontaneous coronary artery dissection
